# Scapular Notching on Kinematic Simulated Range of Motion After Reverse Shoulder Arthroplasty Is Not the Result of Impingement in Adduction

**DOI:** 10.1097/MD.0000000000001615

**Published:** 2015-09-25

**Authors:** Alexandre Lädermann, Boyko Gueorguiev, Caecilia Charbonnier, Bojan V. Stimec, Jean H.D. Fasel, Ivan Zderic, Jennifer Hagen, Gilles Walch

**Affiliations:** From the Division of Orthopaedics and Trauma Surgery, La Tour Hospital, Meyrin (AL); Faculty of Medicine, University of Geneva (AL); Division of Orthopaedics and Trauma Surgery, Department of Surgery, Geneva University Hospitals, Geneva (AL); AO Research Institute Davos, Davos (BG, IZ, JH); Artanim Foundation, Medical Research Department (CC); Faculty of Medicine, Department of Cellular Physiology and Metabolism, Anatomy Sector, University of Geneva, Geneva, Switzerland (BVS, JHF); and Department of Orthopaedics, Shoulder Unit, Santy Orthopaedic Center and Jean Mermoz Hospital GDS, Lyon, France (GW).

## Abstract

Impingement after reverse shoulder arthroplasty (RSA) is believed to occur from repetitive contact in adduction between the humeral component and the inferior scapular pillar. The primary purpose of this biomechanical study was to confirm the presence of different types of impingement and to examine which daily-life movements are responsible for them. A secondary aim was to provide recommendations on the type of components that would best minimize notching and loss of range of motion (ROM).

The study included 12 fresh frozen shoulder specimens; each had a computed tomography (CT) image of the entire scapula and humerus in order to acquire topological information of the bones before RSA implantation. Cyclic tests were run postimplantation with 3 shoulders in each modalities. To quantify bone loss due to impingement, 3-dimensional anatomical models of the scapula were reconstructed from the CT scans and compared to their intact states.

We found 8 bony impingements in 7 specimens: 2 at the lateral acromion, 1 at the inferior acromion, 4 scapular notching, and 1 with the glenoid resulting to wear at the 3:00 to 6:00 clock-face position. Impingements occurred in all kinds of tested motions, except for the internal/external rotation at 90° of abduction. The 3 specimens tested in abduction/adduction presented bone loss on the acromion side only. Scapular notching was noted in flexion/extension and in internal/external rotation at 0° of abduction. The humeral polyethylene liner was worn in 2 specimens—1 at the 6:00 to 8:00 clock-face position during internal/external rotation at 0° of abduction and 1 at the 4:00 clock-face position during flexion/extension.

The present study revealed that 2 types of impingement interactions coexist and correspond to a frank abutment or lead to a scapular notching (friction-type impingement). Scapular notching seems to be caused by more movements or combination of movements than previously considered, and in particular by movements of flexion/extension and internal/external rotation with the arm at the side. Polyethylene cups with a notch between 3 and 9 o’clock and lower neck-shaft angle (145° or 135°) may play an important role in postoperative ROM limiting scapular notching.

## Abstract 2

Supplemental Digital Content is available in the text

## INTRODUCTION

Reverse shoulder arthroplasty (RSA) transforms a spinning joint into a hinge joint. The latter configuration can lead to impingements that are dependent on the spatial positioning of the arm, as well as on the positioning of the prosthetic components. Scapular notching after RSA is the most common complication.^1^ It is believed that this occurs from repetitive contact in adduction between the humeral component and the inferior scapular pillar.^2,3^ However, a recent study demonstrated that contact could occur with other parts of the scapular neck, glenoid, and acromion.^4,5^ Impingements are conditioned by preoperative factors such as erosion of the upper glenoid bone,^6,7^ design of the prosthesis (glenoid lateralization or eccentric glenoid),^8–11^ and surgery-related factors, such as craniocaudal positioning of the glenosphere.^12,13^ These factors can lead to polyethylene debris resulting in the osteolytic reaction,^1^ true bone loss, or to limited postoperative range of motion (ROM). All of these complications can adversely affect the clinical outcome.^3,14^

We hypothesize that 2 kinds of impingement co-exist after RSA. First, an abutment-type would cause limited bony compaction and polyethylene wear, but also a restricted ROM. This impingement would occur in abduction, adduction, and maximal flexion. Second, a friction-type impingement that would occur during rotation, mid-range flexion, and extension.

The primary purposes of this biomechanical study were to confirm the presence of different types of impingement, to quantify the rate of bone loss, and to examine which daily-life movements are responsible for them. A secondary aim was to provide recommendations on the type of components that would best minimize notching and loss of ROM.

## MATERIALS AND METHODS

The study included 12 fresh frozen (−20 °C) shoulder specimens from 7 deceased donors (6 women, 1 man) with native scapula and humerus. All donors gave their informed consent within the donation of an anatomical gift statement during their lifetime. As the data do not contain personal identifiers (anonymous biological material), this research does not require review by an IRB under our federal law (Human Research Act 810.30, HRA). The mean age was 84.5 years (range, 56–101 years). All frozen shoulders had a computed tomography (CT) image of the entire scapula and humerus of 0.63 mm slice resolution (Siemens SOMATOM Emotion 6, Siemens AG Medical Solutions, Forchheim, Germany) to acquire topological information of the bones before implantation.

Specimens were thawed at room temperature for 24 h before prosthesis implantation and biomechanical testing. The surgical technique was standard through a deltopectoral approach.^15^ Delta reverse prostheses (Delta Xtend ™, DePuy International Ltd, Leeds, UK) were implanted by 1 experienced surgeon (AL, blinded for review purpose) in all specimens. The humeral cut of the Delta positioned the humeral component at the level of the top of the humeral head, as previously recommended.^16^ A circular baseplate was implanted at the inferior edge of the glenoid surface and a 38 mm glenosphere was placed over the baseplate. The stem size was 8 mm in 3 cases and 10 mm in 4 cases, and all epiphysis were of size 1. The recommended retroversion of 20^17–19^ was used for all humeral components. The humeral stems were all cemented. Nonconstrained standard humeral polyethylene liners of 3 mm were then impacted on the humeral components to restore humeral and arm length.^16,20,21^ The soft tissue and bony architecture of the scapula and humerus were left intact.

**FIGURE 1:**
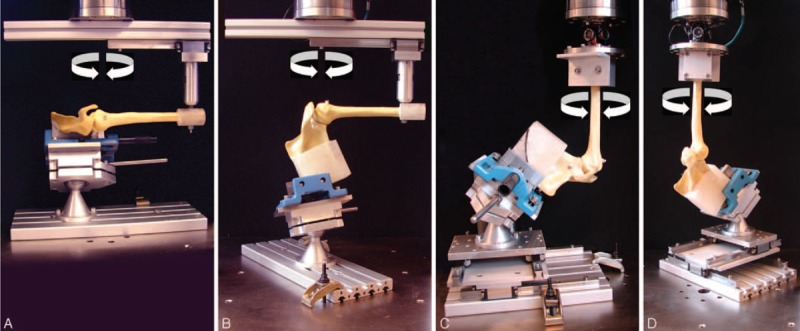
Test setup showing a model of synthetic shoulder mounted for biomechanical testing in abduction/adduction (A), flextion/extension (B), internal/external rotation at 0° abduction (C), and at 90° abduction (D). The human cadaveric specimens were tested in the same fashion.

The inferior (distal) parts of the scapula and humerus were separately embedded in polymethylmethacrylate (PMMA, SCS Beracryl D28, Swiss Composite, Jegenstorf, Switzerland) and attached to a testing machine (MTS 858 Bionix, MTS Systems Corp, Minneapolis, MN) with a 25 kN/200 Nm load cell in a test setup, as shown in Figure 1.

The test setup was realized in 4 variations, allowing cyclical testing through the rotational sinusoidal movements of the machine actuator to test each specimen in one of the following 4 modalities: abduction/adduction, flexion/extension, or internal/external rotation at 0° and 90° of abduction. For specimen's testing in abduction/adduction and flexion/extension, the distal embedded part of the humerus was attached to the machine actuator via a sleigh, able to glide perpendicularly to the vertical actuator axis, whereas the inferior part of the scapula was fixed to the machine base via a vice with adjustable inclination (Figure 1A–B). A cardan joint, connecting the distal humeral part to the machine actuator, and an XY-table, inserted between the vice and the machine base, modified/facilitated the setup for testing in internal/external rotation at 0° and 90° abduction (Figure 1C–D). The scapula and humerus were zeroed to a rest position, according to van Andel et al,^22^ and using the recommended bone coordinates systems.^23^ The zero of abduction/adduction and flexion/extension was set when the thoraco-humeral elevation angle was equal to zero. The zero for rotation was set with the forearm in the coronal plane. Each specimen was tested (in the respective modality) >73,000 cycles, representing 100 movements per day over a period of 2 years. The cyclic test was operated in angle control (of the machine actuator) and consisted of 3 loading steps, split by 5000 and 35,000 cycles and with a constant ROM each. By bringing the shoulder through a full arc of motion at the beginning of cyclic testing, and then after 5000, 35,000, and 73,000 cycles (end of the test), the ROM of the specimen in the respective trial and step was defined manually (and recorded) once reaching ± 5 Nm torque in each rotational direction of the machine actuator; this limit was determined from pilot tests and set to minimize undue tissue fatigue.

**FIGURE 2:**
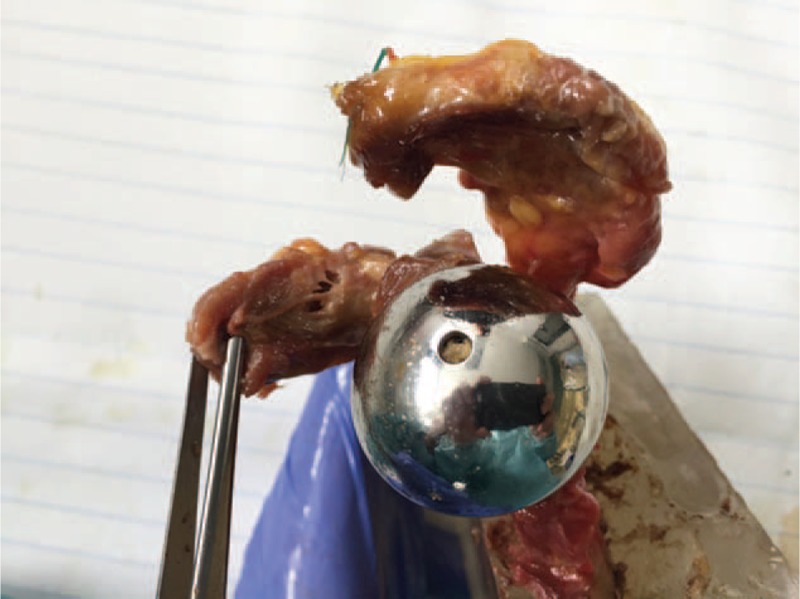
Lateral view of a right shoulder after dissection. The soft tissues were removed and the fracture of the coracoid process was clinically observed in this case.

Three specimens were tested in each of the 4 modalities (12 specimens in total). The purpose of cyclic testing was to observe, for each prosthetic configuration, what types of impingement occurred in daily activities, and whether the ROMs increase as wear accumulated. After 73,000 cycles, dissection was performed. The soft tissues of the glenoid, scapular neck and spine, coracoid, acromion, and the prosthetic components were removed (Figure 2). Bony impingement (erosion, impaction), polyethylene wear, fatigue fracture of the acromion, coracoids, or scapular spine were clinically observed and reported. A new CT scan of the entire scapula was also performed using the imaging parameters described previously.

**FIGURE 3:**
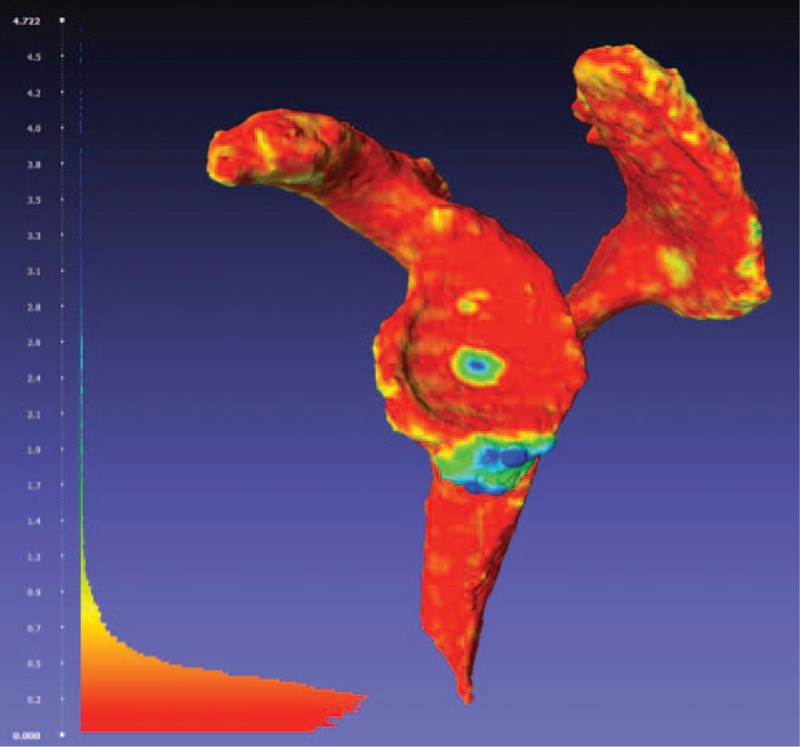
Visualization of the point-to-mesh distances on the *MBI* model. The colors represent the variations of distance between the *MBI* and *MAE* models. The blue color denotes the zones of maximum distance (= maximum bone loss or wear). Note: the *MAE* model which is superposed on the *MBI* model is not shown for clarity. MAE = model after explantation; MBI = model before implantation.

To quantify bone loss due to impingement, three-dimensional (3D) anatomical models of the scapula were reconstructed from the CT scans using Mimics software version 17.0 (Materialize NV, Leuven, Belgium). The 3D CT images were segmented by a thresholding technique to extract bone contours automatically and by manual segmentation for contours filling and local corrections. Two scapula bone models were thus obtained for each specimen: one model before implantation (*MBI*) and one model after explantation (*MAE*). No smoothing or topological modification of the meshes was performed after 3D reconstruction. To compare the two models, *MBI* and *MAE* were cut to retain the region of interest (glenoid, inferior scapular pillar, acromion, and coracoid) and registered together using the Iterative Closest Point algorithm.^24^ To quantify the geometric difference between the two models, the closest point on the *MAE* mesh was computed for each vertex of the *MBI* mesh and the distance calculated. A color scale was used to map the variations of distance on the *MBI* surface, with the blue color denoting the zones of maximum distance (= maximum bone loss or wear) and other colors denoting the zones of decreased distance (Figure 3). Moreover, the surface area of each damaged zone was measured in 3D and expressed in millimeters. The location of the damaged zone was also reported and compared to the clinical observations.

### Statistical Analysis

Statistical evaluation was performed by the use of software package R, version 3.1.1. Descriptive analysis consisted of frequencies and percentages for discrete data and means and standard deviations for continuous data. ROM of the specimens in all 4 modalities during the cyclic biomechanical testing was computed together with the prevalence of bony impingement, polyethylene wear, and fatigue fracture. The surface area and the corresponding maximum distance of the damaged zones were also reported for each impingement. Cohen's kappa coefficient (*К*) was calculated to assess the interobserver agreement between the clinical observations and the topological 3D analysis.

## RESULTS

The results from the evaluation of the ROM in all 4 modalities during the cyclic biomechanical testing are given in Table 1. A progressive increase during the cyclic test was observed for all modalities and directions.

**TABLE 1:**
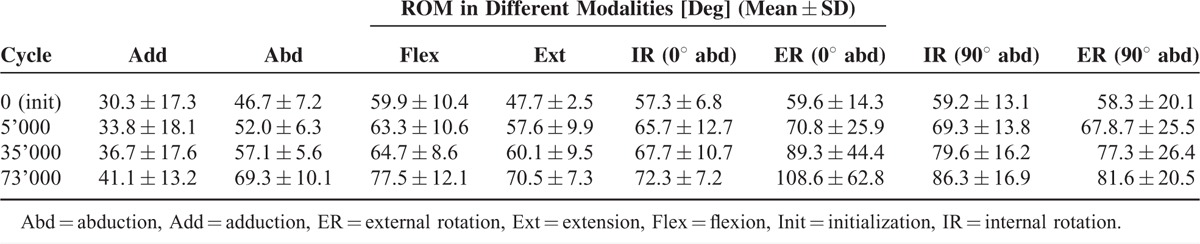
ROM Among the Subjected Specimens in the 4 Modalities During the Cyclic Biomechanical Testing

The *К* value for interobserver agreement between observations made at dissection and the ones issued from the topological 3D analysis was 0.93, representing almost perfect agreement.^25^

**FIGURE 4:**
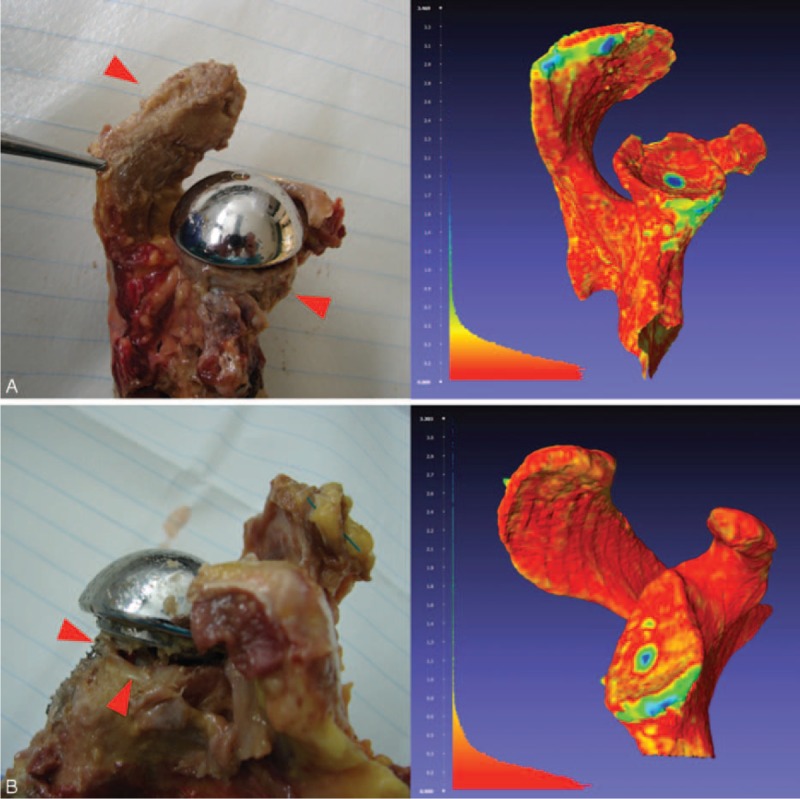
(A) Impingement with lateral acromion and scapular notching (arrows). (B) Glenoid bone loss at the 3:00 to 6:00 clock-face position (arrows). Left: photographs taken at dissection. Right: visualization of the point-to-mesh distances on the *MBI* model as described above MBI = model before implantation.

We found 8 bony erosions in 7 specimens (Table 2): 2 at the lateral acromion, 1 at the inferior acromion, 4 scapular notching, and 1 with the glenoid resulting to wear at the 3:00 to 6:00 clock-face position. Figure 4 represents 2 different bone impingements found in the study. Impingements occurred in all tested motions, except for the internal/external rotation at 90° of abduction. The 3 specimens tested in abduction/adduction presented bone loss on the acromion side only (Table 2). Scapular notching was mainly noted in flexion/extension and in internal/external rotation at 0° of abduction. The humeral polyethylene liner was worn in 2 specimens—1 at the 6:00 to 8:00 clock-face position during internal/external rotation at 0° of abduction and 1 at the 4:00 clock-face position during flexion/extension. Two compressions or fatigue fractures of the coracoid were observed in 2 specimens during flexion/extension.

**TABLE 2:**
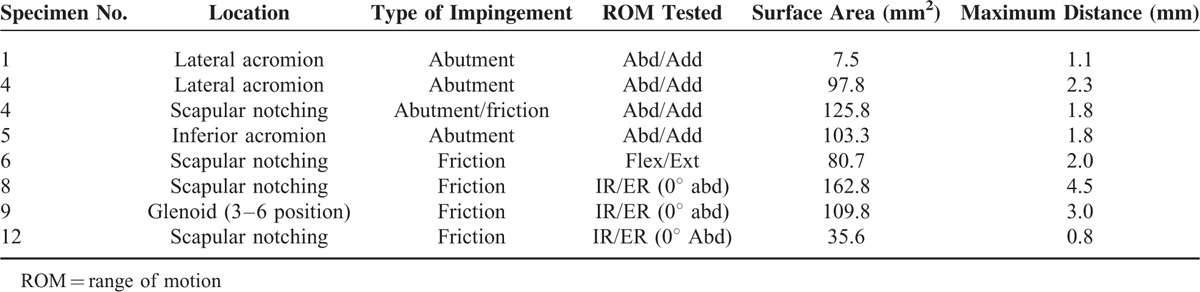
Bony Impingements With their Location, the ROM Tested, the Surface Area, and the Corresponding Maximum Distance of the Damaged Zones

## DISCUSSION

The glenohumeral joint has the largest ROM among all diarthrodial joints. One of the goals of shoulder prosthesis implantations, as for many other total joint implant systems, is to restore the native function and consequently obtain an impingement free arc-of-motion. Design of Grammont RSA produced secondary changes in joint biomechanics.^26^ One such change, the medialization of the center of rotation, is believed to be responsible for impingement of the medial border of the humeral component on the scapular neck when the arm is adducted.^13^ Anterior and posterior notching have also been attributed to impingement with the prosthesis in internal and external rotations, respectively.^14^ The prevalence of scapular notching is high, observed in 88% in the series of Mélis et al.^1^ Repetitive contact between polyethylene and bone may result in polyethylene wear debris.^27^

The present study revealed that 2 types of impingement interactions coexist, confirming our hypothesis. We proposed that impingement could correspond to a frank abutment with no possibilities to continue movement (compression or fatigue fracture, Figure 4A and movie 1, Video 1 Lateral view of a right shoulder. Note the abutment-type impingement between the greater tuberosity and the acromion, http://links.lww.com/MD/A425), or lead to a scapular notching when the humeral socket engages the glenoid circumferentially (friction-type impingement, Figure 4B and movie 2, Video 2 Anterior view of a left shoulder. The polyethylene engages the glenoid circumferentially [friction-type impingement] and causes scapular notching by movements of internal/external rotations with the arm at the side, http://links.lww.com/MD/A426).

The abutment-type impingement seems to limit ROM in abduction and flexion with a contact zone located on the lateral acromion or the coracoid process. Lädermann et al with a 3-dimensional computer model of RSA previously described such an impingement of the proximal humerus with the superior glenoid fossa, the acromion in abduction, and in external rotation at 90° of abduction.^28^ Impingement within the latter modality was likely not demonstrated in the present study due to the use of nonlateralized glenoid component and 155° neck-shaft angle.^28^ This repetitive contact between the humerus and the scapula might be responsible for compression or fatigue fracture of the acromion or coracoid process with other implant designs. This could be another factor, in addition to deltoid retentioning^20^ and osteoporosis, responsible for postoperative acromial fracture or migration.

Contrarily, some impingements seem to be related to a friction of the polyethylene against the bone in flexion, extension, and during rotation (friction-type impingement, movie 2). Such an impingement might result in millimeters of bone wear, but would still allow continuation of movement. We believe that these repetitive phenomena might potentially lead, with time, to progressive bony and polyethylene abrasion without limiting ROM and could radiologically explain rapid apparition of scapular notching. They are the results of multiple movements (adduction, rotations, and extension) and not the consequence of a simple contact with the pillar in adduction with the arm at the side as previously believed. Those findings may explain why patients with RSA continue to experience increase in ROM over months.^29^

Previous studies have demonstrated that postoperative active ROM was determined by numerous factors. The type of implant,^5,17,30^ the morphology of the scapula,^31^ and pre-,^32,33^ intra-,^34^ and postoperative^16,21^ soft tissue considerations are known to be contributors. The present study revealed that the type of impingement induced by the reverse design is another key element. As all impingements in adduction, extension, and rotation at 0° of abduction occur between the polyethylene and the scapular neck, it seems thus logical to promote polyethylene cups with a notch between 3 and 9 o’clock, as in other designs (Arrows, SMR, Affinis, etc). Moreover, the results of this study could explain why new humeral shaft designs with lower neck-shaft angle (145° or 135°) may play an important role in postoperative ROM limiting scapular notching.

### Strengths and Limitations

To our knowledge, this is the first study which specifically investigated different types of impingement after RSA. Despite the complexity and the length of testing, we were able to test a consequent sample size of 12 shoulders. This allowed us to analyze all possible motions with multiple morphologies. This is important as changes related to human scapular morphology, such as scapular neck or critical shoulder angles, also impact the tendency towards impingement.^31^ However, the number of specimen did not allow for the comparison of the different sizes of glenospheres. Another limitation of this study is the partial omission of the humeral sided wear. Even if polyethylene liner wear was detected in 1 specimen, it was impossible to accurately quantify with CT scan the humeral bone loss between performance of the humeral cut at the anatomical neck and after necessarily destructive prosthetic and cement removal.

## CONCLUSIONS

Several types of impingement exist in RSA. Scapular notching seems to be caused by more movements or combination of movements than previously considered, and in particular by movements of flexion/extension and internal/external rotations with the arm at the side.
